# Genome-wide association study identifies host genetic variants influencing oral microbiota diversity and metabolic health

**DOI:** 10.1038/s41598-024-65538-8

**Published:** 2024-06-26

**Authors:** Evelina Stankevic, Timo Kern, Dmitrii Borisevich, Casper Sahl Poulsen, Anne Lundager Madsen, Tue Haldor Hansen, Anna Jonsson, Mikkel Schubert, Nikoline Nygaard, Trine Nielsen, Daniel Belstrøm, Tarunveer S. Ahluwalia, Daniel R. Witte, Niels Grarup, Manimozhiyan Arumugam, Oluf Pedersen, Torben Hansen

**Affiliations:** 1 The Novo Nordisk Foundation Center for Basic Metabolic Research, Faculty of Health and Medical Sciences, University of Copenhagen, Copenhagen, Denmark; 2 Medical Department, Zealand University Hospital, Koege, Denmark; 3 Department of Clinical Medicine, University of Copenhagen, Copenhagen, Denmark; 4 Department of Public Health, Aarhus University, Aarhus, Denmark; 5 Steno Diabetes Center Copenhagen, Herlev, Denmark; 6 The Bioinformatics Center, Department of Biology, University of Copenhagen, Copenhagen, Denmark; 7 Department of Odontology, Section for Clinical Oral Microbiology, University of Copenhagen, Copenhagen, Denmark; 8 Steno Diabetes Center Aarhus, Aarhus, Denmark; 9 Center for Clinical Metabolic Research, Herlev-Gentofte University Hospital, Copenhagen, Denmark

**Keywords:** Oral microbiota, Oral health, Metabolic disease, Single nucleotide polymorphism, Microbiome-genome wide association study, Host genetics and microbiota interplay, Genome-wide association studies, Bacterial genomics

## Abstract

The microbial communities of the oral cavity are important elements of oral and systemic health. With emerging evidence highlighting the heritability of oral bacterial microbiota, this study aimed to identify host genome variants that influence oral microbial traits. Using data from 16S rRNA gene amplicon sequencing, we performed genome-wide association studies with univariate and multivariate traits of the salivary microbiota from 610 unrelated adults from the Danish ADDITION-PRO cohort. We identified six single nucleotide polymorphisms (SNPs) in human genomes that showed associations with abundance of bacterial taxa at different taxonomical tiers (*P* < 5 × 10^–8^). Notably, SNP rs17793860 surpassed our study-wide significance threshold (*P* < 1.19 × 10^–9^). Additionally, rs4530093 was linked to bacterial beta diversity (*P* < 5 × 10^–8^). Out of these seven SNPs identified, six exerted effects on metabolic traits, including glycated hemoglobin A1c, triglyceride and high-density lipoprotein cholesterol levels, the risk of type 2 diabetes and stroke. Our findings highlight the impact of specific host SNPs on the composition and diversity of the oral bacterial community. Importantly, our results indicate an intricate interplay between host genetics, the oral microbiota, and metabolic health. We emphasize the need for integrative approaches considering genetic, microbial, and metabolic factors.

## Introduction

The oral cavity harbours several hundred different bacterial species making the oral microbiota one of the most complex microbial communities of the human body^1^. Attached to various surfaces of the mouth, the resident microbes prevent colonization by pathogens and orchestrate the maintenance of oral health together with the immune system of the host^2,3^. Thus, a healthy oral microbiota contributes to symbiotic relationships and resilience whereas a disruption of the homeostasis may increase risk of various local or systemic diseases of the host^3–6^. Most prevalent oral diseases, like gingivitis, periodontitis, and dental caries, have a clear bacterial etiological component^4,5^. In addition, epidemiological studies have indicated that an aberrant oral microbiota may play a role in the pathogenesis of systemic diseases^6^, including cardiovascular disease^7,8^, obesity, type-2-diabetes (T2D), and other components of metabolic syndrome^9,10^.

The mechanisms behind the compositional changes leading to dysbiosis of the oral microbiota are still not fully understood. The basis of the complex structure of the oral microbiota is assembled in the early years of life, and it is modulated by several exogenous and endogenous factors including delivery mode, feeding method, sex, and host genetics^11,12^. Alterations in the individual immune response as well as lifestyle factors, such as diet, oral hygiene and smoking have also been shown to induce dynamic compositional and functional changes of the oral microbiota^13–16^. Moreover, prolonged cohabitation increases the similarity in oral microbiota composition among individuals sharing household^17^.

The first pieces of evidence for associations between the oral microbiota and human host genetics were revealed using targeted approaches of candidate genes. For example, variations within *MHC* genes were reported to increase the susceptibility for caries-associated bacteria^18,19^. However, the extent of the influence of genetics on human oral microbiota is debated, with some authors emphasizing the strong influence of non-genetic factors^20–22^. Recent twin studies have demonstrated that a fraction of the oral microbiota is heritable^22–24^; with a study encompassing 752 twin pairs revealing that the oral microbiota of monozygotic twins is compositionally more alike than that of dizygotic twins^24^. Results from another twin study showed that combined effects of individual host genetic variants explain variation in presence and abundance of certain bacterial species, with the strongest genetic effects identified for *Corynebacterium singular*, *Campylobacter concisus*, *Veillonella rogosae*, and *Saccharibacteria* (TM7) [G-1] bacterium HMT 349^22^.

Despite the emerging interest in the field, genome-wide association studies (GWAS) of oral bacterial traits are sparse and only few associations have been identified reaching genome-wide significance^24–26^. Notably, a metagenome-GWAS (mgGWAS) in a Chinese population, that included 1915 individuals in discovery cohort and 1439 in validation cohort, identified two study-wide significant genetic loci rs4911713 and rs36186689, that associated with an unknown species (*F0422 uSGB392*) from the *Veillonellaceae* family and with the genus *Eggerthia*, respectively^26^. A subsequent study of the same cohort also discovered 11 sex-specific genetic variations influencing the composition of salivary microbiota and found indications supportive of a causal link between several bacterial species and the risk of T2D^12^.

Here, we provide novel insights into the interaction between oral bacterial microbiota and host genome variation using GWAS of the oral microbiota from an unrelated sample of the Danish ADDITION-PRO cohort^27^, consisting of older middle-aged individuals at various levels of risk of developing T2D.

## Results

In total, 610 participants with information about the oral microbiota and genotype data were included in the analysis. Descriptive characteristics of the participants are summarised in Table 1 (Table 1). We have tested the association between 43 univariate and one multivariate oral bacterial features and 5,195,096 and 5,374,467 human autosomal variants, respectively.

**Table 1:** Characteristics of the study participants (N = 610) grouped by sex. Mean (SD).

	Total	Women	Men
Participants (n)	610	277	333
Age (years)	67.6 (6.0)	68.3 (5.7)	67.0 (6.2)
BMI (kg/m^2^)	26.9 (4.2)	26.5 (4.5)	27.3 (3.8)
Smoking status			
* ex-smoker*	104 (17.0)	38 (13.7)	66 (19.8)
* non-smoker*	289 (47.4)	111 (40.1)	178 (53.5)
* Smoker*	217 (35.6)	128 (46.2)	89 (26.7)
Glycemic state			
* NGT*	343 (56.2)	170 (61.4)	173 (52.0)
* IFG*	98 (16.1)	34 (12.3)	64 (19.2)
* IGT*	48 (7.9)	27 (9.7)	21 (6.3)
* IFG & IGT*	51 (8.4)	25 (9.0)	26 (7.8)
* screen detected T2DM*	57 (9.3)	16 (5.8)	41 (12.3)
* T2D*	11 (1.8)	4 (1.4)	7 (2.1)
* unclassified*	2 (0.3)	1 (0.4)	1 (0.3)
Plasma HbA1c (%)	5.7 (0.4)	5.7 (0.4)	5.7 (0.5)
Fasting plasma glucose (mmol/l)	6.0 (0.8)	5.8 (0.6)	6.1 (0.8)

Categorical variables are summarized by count (percent). Abbreviations: BMI body mass index, NGF normal glucose tolerant, IFG impaired fasting glucose, IGT impaired glucose tolerance, T2D type 2 diabetes. HbA1c glycated hemoglobin A1c.

### Host genetic variants associated with bacterial beta diversity

**Figure 1:**
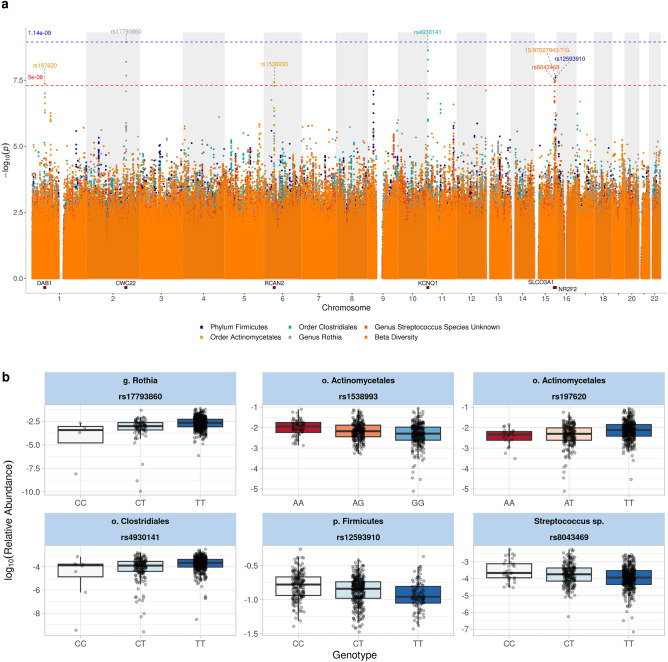
Results from the GWAS. (**a**) Manhattan plot representing results from the GWAS on oral microbial features. The red line represents the genome-wide significance level (*P* = 5 × 10^–8^) and the blue line represents study-wide significance level (*P* = 5 × 10^–8^ / 44). The rsID and nearest gene annotation is identified and included using topR R package. The color-coding represents 6/44 bacterial features that had variants reaching genome-wide significance level (*P* < 5 × 10^–8^). (**b**) Box plots representing the genetic effect of SNPs identified to significantly associate with abundance of bacterial taxa. The box plot displays the lower quartile, median, and upper quartile values within a box.

Using a distance-based F-test^28^ based on the Bray–Curtis dissimilarity matrix, we investigated associations between individual genome variants and overall oral bacterial community composition (beta diversity). Two variants on chromosome 15 were significantly associated to beta diversity (*P* < 5 × 10^–8^) (Fig. 1a). The effect allele of rs4530093 and the effect allele of rs11073493 were in high linkage disequilibrium (LD) (r^2^ > 0.8) indicating one independent signal close to *RN7SKP181*.

### Host genetic variants associated with individual bacterial taxa

**Figure 2:**
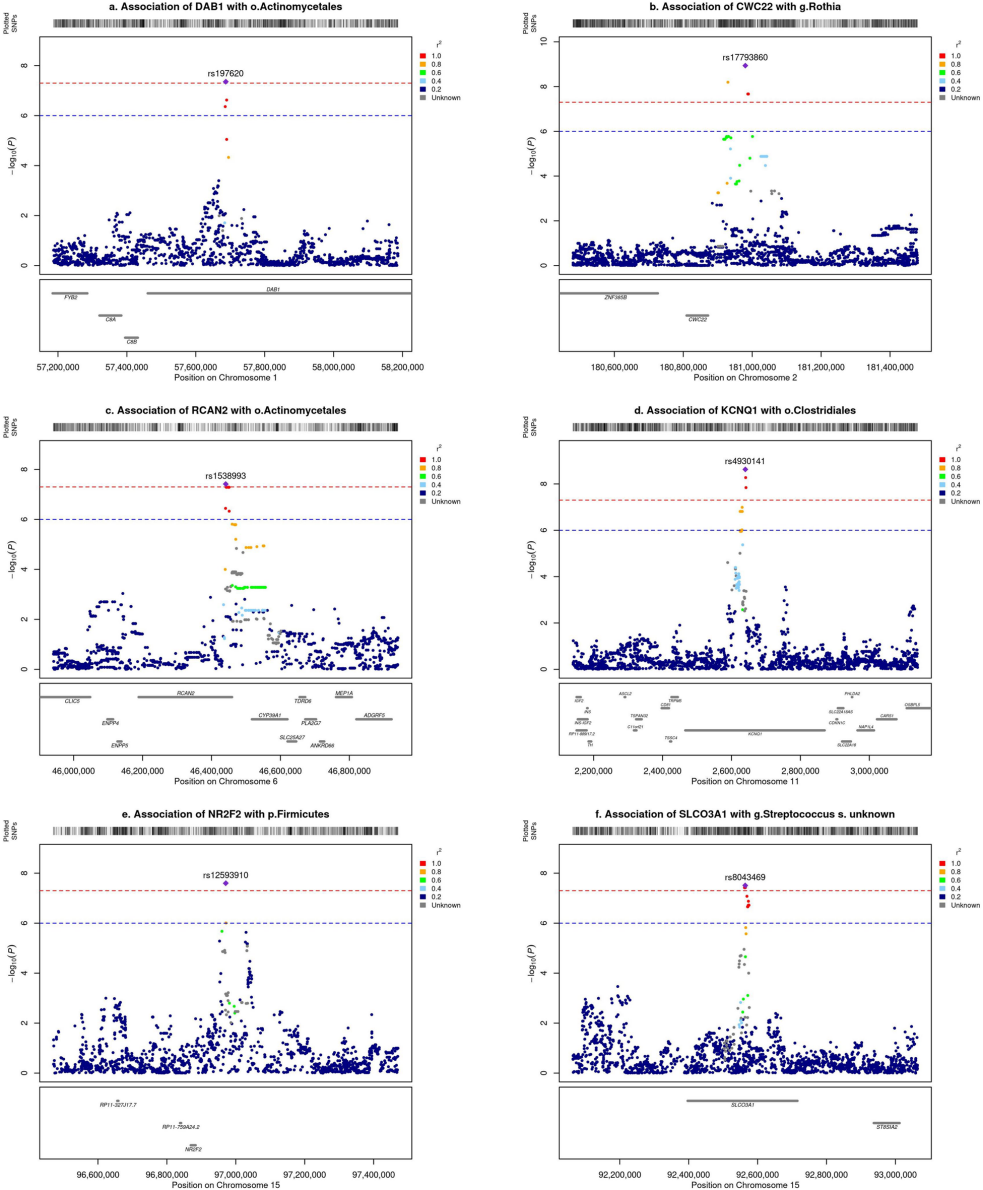
Locus Zoom plots showing regional association with univariate bacterial features*.* (**a**) *DAB1*, chromosome 1: 57687078 (rs197620); *P* = 4.41 × 10^−8^, (**b**) *CWC22*, chromosome 2:180979913 (rs17793860); *P* = 1.16 × 10^−9^, (**c**) *RCAN2*, chromosome 6:46441428 (rs1538993); *P* = 3.86 × 10^−8^, (**d**) *KCNQ1*, chromosome 11:2639985 (rs4930141); *P* = 2.37 × 10^−9^, (**e**) *NR2F2* chromosome 15:96970958 (rs12593910); *P* = 2.53 × 10^−8^, (**f**) *SLCO3A1* chromosome 15:92564182 (rs8043469); *P* = 3.10 × 10^−8^.

In total, six loci showed genome-wide significant associations (*P* < 5 × 10^–8^) with univariate bacterial features, out of which one locus, rs17793860, reached study-wide significant association (*P* < 1.19 × 10^–9^) (Fig. 1a, Table 2). The effect of genotype on the abundance of bacterial taxa is visualised in Fig. 1B. Most of the associations were found at the taxonomical level order (N = 3); while one association each was found at phylum, class and species level, respectively. Two genetic loci were associated with *Actinomycetales* order (rs1538993; *RCAN2* and rs197620; *DAB1*) (Fig. 2a–c) and one with genus *Rothia* from *Actinomycetales* order (rs17793860; *CWC22*) (Fig. 2b). The *KCNQ1* locus (rs4930141) was associated with *Clostridiales* order (Fig. 2d**, **Table 2), the *NR2F2* locus (rs12593910) was associated with phylum *Firmicutes* (Fig. 2e), and the *SLCO3A1* locus was associated with abundance of an unknown *Streptococcus* species (Fig. 2f).

**Table 2:** Results summary of GWAS for univariate bacterial features.

Chromosome	Position	P	Effect Size	A1	A2	Standard Error	rsID	Taxon Name	Taxon Level	Gene Name	Gene BioType
1	57687078	4.41E−08	− 0.393	T	A	0.071	rs197620	*Bacteria;Actinobacteria;Actinobacteria;Actinomycetales*	Order	DAB1	intron_variant
2	180979913	1.16E−09	− 0.653	T	C	0.106	rs17793860	*Bacteria;Actinobacteria;Actinobacteria;Actinomycetales;Micrococcaceae;Rothia*	Genus	CWC22	intergenic_variant
6	46441428	3.86E−08	− 0.334	A	G	0.060	rs1538993	*Bacteria;Actinobacteria;Actinobacteria;Actinomycetales*	Order	RCAN2	intron_variant
11	2639985	2.37E−09	− 0.497	T	C	0.082	rs4930141	*Bacteria;Firmicutes;Clostridia;Clostridiales*	Order	KCNQ1	intron_variant
15	96970958	2.53E−08	− 0.337	C	T	0.060	rs12593910	*Bacteria;Firmicutes*	Phylum	NR2F2	intergenic_variant
15	92564182	3.1E−08	0.372	T	C	0.066	rs8043469	*Bacteria;Firmicutes;Bacilli;Lactobacillales;Streptococcaceae;Streptococcus;ASV0009*	Species	SLCO3A1	intron_variant

The effect size and standard error are rounded to 3 digits. The effect size is calculated for the A2 allele.

#### Single-genetic variant associations with metabolic traits

**Figure 3:**
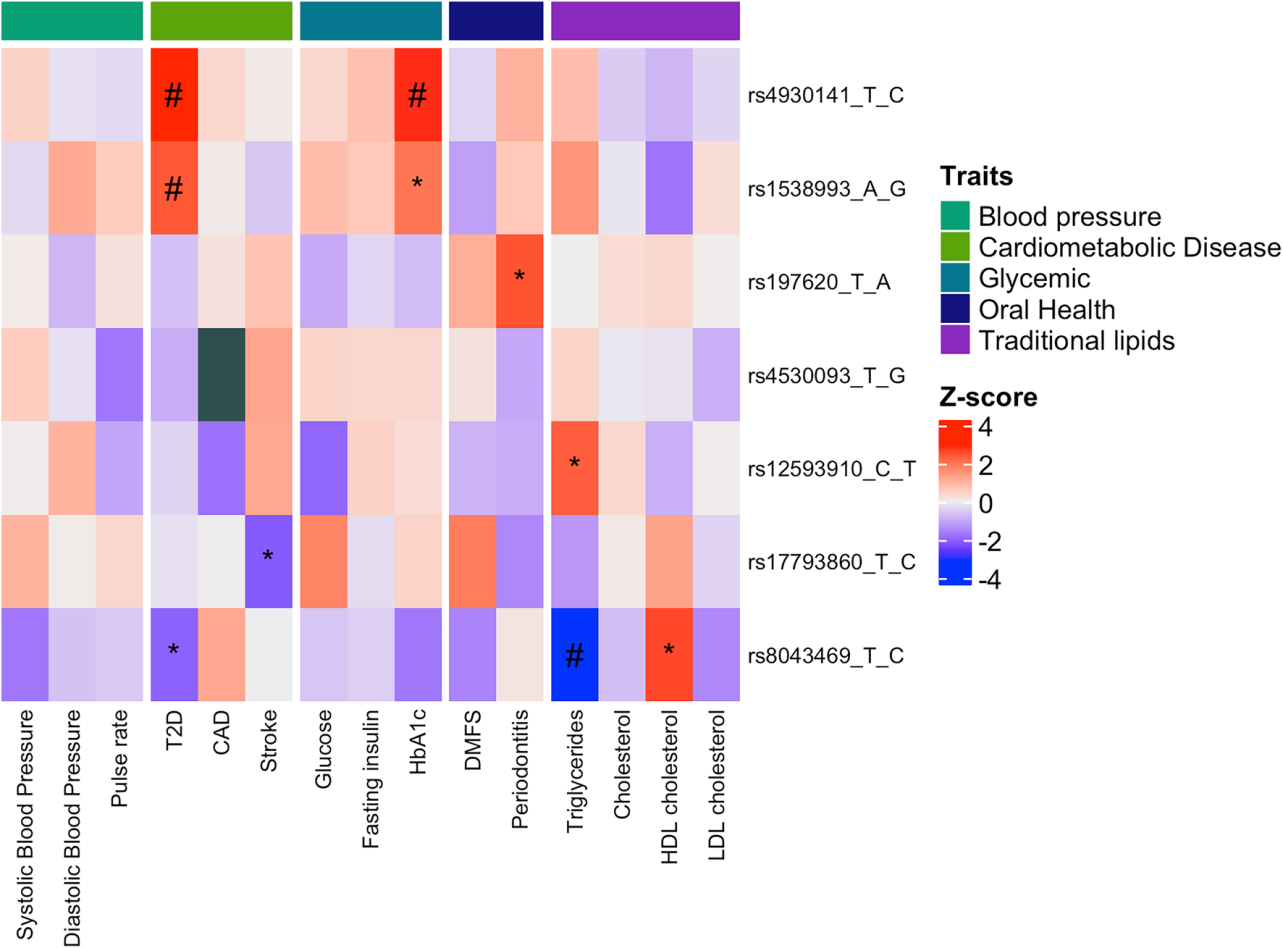
Single variant associations with metabolic, anthropometric and lifestyle traits. For each lead variant from GWAS analysis of univariate and multivariate microbial features (y axis), Z scores (aligned to effect (alternative) allele) were obtained from publicly available GWAS (Table S3) for metabolic, anthropometric and lifestyle traits (x axis). Heatmap reflects the direction and magnitude of the Z score for the association between SNP and common traits, with red indicating positive and blue indicating negative associations. Grey squares indicate that variant was not available in the summary statistics for the given trait. *Indicates *P* value < 0.05, # indicates FDR adjusted *P* value < 0.05. Abbreviations: T2D, type-2 diabetes; CAD, coronary artery disease; HbA1c, glycated hemoglobin A1c; DMFS, decayed, missing, filled surfaces; HDL, high-density lipoprotein; LDL, low-density lipoprotein.

**Figure 4:**
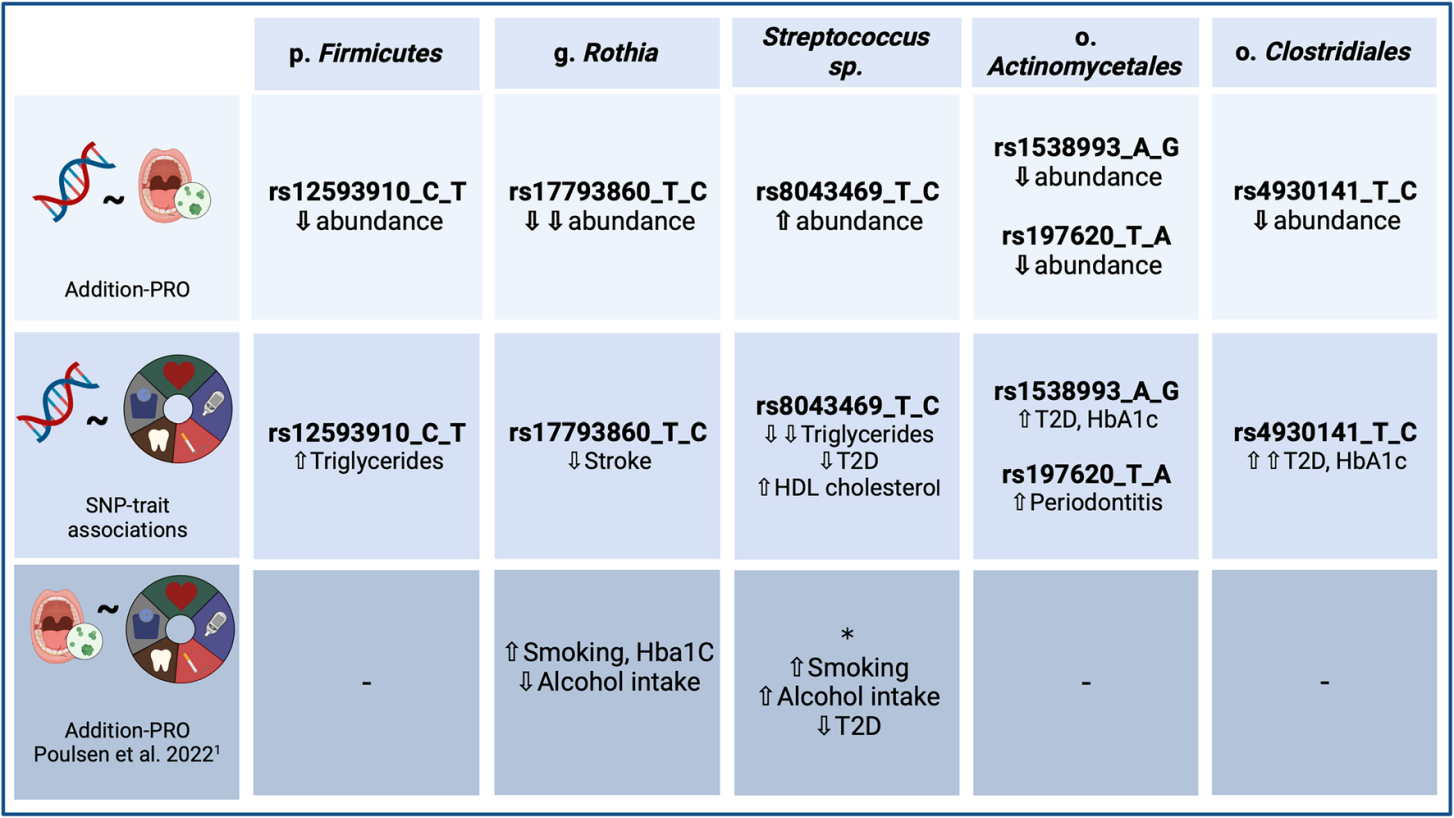
Overview of study results. Graphical table represents summary of the study findings and integration with already published data. The first row represents the results of microbiome-genome wide association study, with identified associations between variants and the abundance of bacterial taxa in saliva. The second row summarizes how the identified variants associate with metabolic traits. The third row integrates observational findings from previously published study on associations between oral microbiome composition and lifestyle factors (^1^Poulsen et al. Association of general health and lifestyle factors with the salivary microbiota—Lessons learned from the ADDITION-PRO cohort. Front Cell Infect Microbiol. 2022 Nov 16;12:1055117. PMID: 36467723). One arrow in bold represents the association and direction of the effect from GWAS, reaching genome-wide significance level at *P* < 5 × 10^–8^, two arrows in bold represent study-wide significant association at *P* < 1.19 × 10^–9^. One arrow represents the direction and association at *P* value < 0.05, two arrows represent association at *P* value < 0.05 after correction for multiple testing (FDR). Asterisk * represents association of genus *Streptococcus*. Figure created with Biorender. Abbreviations: p., phylum; g., genus; sp., unknown single species; o., order; SNP, single nucleotide polymorphism; T2D, type-2 diabetes; HDL, high-density lipoprotein; HbA1c, glycated hemoglobin A1c.

We queried publicly available GWAS datasets for associations involving the six microbiome-associated variants (Figs. 3, 4). The rs17793860-C allele of the *CWC22* locus that associates with decreased abundance of genus *Rothia,* was also associated with decreased risk of stroke. The rs8043469-C allele of *SLCO3A1* locus that associates with increased abundance of unknown species from genus *Streptococcus,* was associated with lower triglyceride levels, lower risk of T2D, and higher HDL-cholesterol levels. The rs1538993-G allele of the *RCAN2* locus that associates decreased abundance of order *Actinomycetales,* was associated with higher HbA1c levels and risk of T2D. The rs4930141-C allele of the *KCNQ1* locus that associates with decreased abundance of order *Clostridiales,* was strongly associated with higher HbA1c levels and risk of T2D. The rs12593910-T allele of the *NR2F2* locus that associates with decreased abundance of phylum *Firmicutes,* was positively associated with triglyceride levels. The rs197620-A allele of *DAB1* locus that associated with decreased abundance of order *Actinomycetales,* associated with higher risk of periodontitis. No significant associations with publicly available GWAS datasets were identified for rs4530093 of *RN7SKP181* (Figs. 1b, 3).

## Discussion

This study aimed to identify host genome variants that influence oral microbial traits. We report six genome-wide significant associations of genome variants with abundance and prevalence of oral bacterial taxa, and one with overall bacterial community composition as assessed by Bray–Curtis dissimilarity in a high risk pre-diabetes cohort. The identified host genome variants also exhibit effects on metabolic traits, indicating potential implications for metabolic disease (Fig. 4).

The overall impact of genetic factors on the interpersonal variability of the human gut and oral microbiota composition in relation to non-genetic factors is debated^20,23,24^. While genome-wide analysis approaches targeting the oral microbiota are scarce, there is a growing interest in understanding how host genetics influence the composition of oral microbes. In a recent mgGWAS, Liu et al. identified 455 independent associations for the salivary microbiota in 1,915 Chinese individuals with two study-wide significant associations and one genetic variant (rs73243848) influencing the beta diversity of the saliva microbiota^26^. Our study was unable to replicate prior findings, possibly due to considerable differences in sequencing methods used, subsequent data processing, including taxonomical classification of bacteria, and the statistical methods. These differences make direct comparison between studies challenging.

Demmitt et al. performed a GWAS analysis of six bacterial traits showing the highest heritability as assessed by ACE-twin modelling and by Genome Complex Trait Analysis (GCTA)^29^ for 818 unrelated individuals. An unnamed species of *Granulicatella* (55.8% heritable, CI 0.28–0.63) and principal coordinate 2 (PCo2) of Bray–Curtis dissimilarity (46.3% heritable, CI 0.23–0.55) have been reported with the highest heritability of oral bacterial traits based on analyses of twins. Further, Demmitt et al. have identified an association of a variant located in the gene *LIN7A* with PCo3 of unweighted UniFrac of oral microbiota^24^. Likewise, our findings suggest an effect of host genome variants on bacterial beta diversity. But instead of performing an ordination of the dissimilarity matrix in order to condense the variation to lower-dimensional representation, we have used a distance-based F-test (DBF-test)^28^ directly on the pairwise dissimilarity matrix. Thus, we were able to capture variation that extends beyond what is typically displayed by the first major ordination axes^28^. Using this approach, we identified one independent signal in the locus of the gene *RN7SKP181.*

The abundance of the order *Clostridiales* was associated with genetic variant rs4930141 in the *KCNQ1* locus that encodes a potassium channel that is important for cardiac repolarization^30^. Notably, variations in the *KCNQ1* have been found to associate with T2D risk^31^. Variation in rs4930141, which associated with decreased abundance of order *Clostridiales* in saliva*,* also associated with increased risk of T2D. Interestingly, *Clostridiales sp.* have also been found to be differentially decreased in the gut microbiota of adults with type-1 diabetes^32^. The abundance of *Firmicutes* was inversely associated with the effect allele of rs12593910, a variant located near the *NR2F2*. In a seminal study by Zaura et al., the phylum *Firmicutes* was identified as one of the core microbes of the healthy oral cavity^33^. Notably, in our study the same effect allele of rs12593910 also associates with elevated triglyceride levels, suggesting that a decrease in *Firmicutes* abundance and an increase in triglyceride levels may contribute to dysmetabolism, possibly through overlapping genetic factors. While these associations suggest a relationship between oral microbiota and human metabolic health, it is essential to validate the findings and establish causation.

Several associations were identified with variations in the host genome and members of the commensal *Actinobacteria* family; including two independent signals, rs1538993 and rs197620, that were linked with a lower abundance of order *Actinomycetales*, and rs17793860, linked with with lower abundance of genus *Rothia*. In addition, the genetic variant rs197620 was associated with higher risk of periodontitis and the genetic variant rs17793860 was associated with lower risk of stroke. In a recent study from our group, Poulsen et al. showed a positive association between abundance of *Rothia* and glycosylated hemoglobin levels^34^. Other investigators showed that abundance of *Rothia* associate to childhood dental caries^23^, and history of colorectal cancer^35^. While some of the species within the *Rothia* genus are typically characterized as benign, others are recognized as opportunistic pathogens, playing a putative role in oral diseases^36,37^ and in lung diseases^38^.

16S rRNA gene amplicon-based sequencing allows a resolution that is reliable only down to the genus level. This hampers the possibility of identifying or replicating already identified associations on species or strain level. One such example is the variation in rs8043469, associated with increased abundance of a species within the *Streptococcus* genus, and a better metabolic profile, including lower triglyceride levels, lower risk of T2D and higher HDL cholesterol levels. Given that *Streptococcus* represents a dominant genus in both healthy and diseased oral microbiota^39^, achieving species or strain-level resolution becomes imperative for the accurate interpretation of such discoveries.

Bacterial taxa, identified to associate with host genetic variants in our study, are involved in specific metabolic pathways that relate to oral and systemic health. For example, phylum *Firmicutes* that includes genus *Streptococcus*, are the main drivers of carbohydrate metabolism in the oral cavity, and certain *Streptococcus* species are involved in biofilm pathogenicity^40,41^. On the other hand, genus *Rothia* is mainly known for its capacity to perform nitrate reduction, which is a crucial biochemical process that contributes to production of nitric oxide (NO). NO is a key signalling molecule that has a central role in blood pressure regulation^42^. In our study, the findings are conflicting—we find that the variant, that associates with decreased abundance of *Rothia* genus, also associates with decreased risk of stroke. This highlights the limitation of the inability to resolve taxonomical rank beyond the genus level. The ability to draw precise functional conclusions is hampered, making it challenging to determine the specific metabolic pathways or microbial activities that might be influenced in different conditions.

Our study has limitations. The statistical power to observe true positive associations is dependent on different factors: first, the number of study participants, and second, the number of tests, which is determined by the number of independent genetic variants and the number of bacterial microbiota traits^43^. To mitigate this, we used clustering approaches reducing the number of associations performed, considering the relatively low sample size (N = 610). Despite the untargeted approach, we have also incorporated multiple testing corrections to mitigate the risk of false positives.

The ADDITION-PRO population consists of older middle aged Danish individuals at various levels of risk of developing T2D. Considering the age of the participants and the different glycemic states, it can be assumed that the health-related non-genetic phenotypes may have affected the oral bacterial composition. Consequently, due to the cohort specific phenotypes, our findings may not be generalizable to other populations. To overcome these limitations, large-scale studies across diverse ethnicities and phenotypes are warranted. Another limiting factor is the large impact of non-genetic factors on oral microbiota. In a previous study conducted within the same population, we identified that among others, glycemic status, sex, smoking, and weekly alcohol intake significantly influenced the composition of salivary microbiota. Therefore, in our current statistical analyses, we adjusted for the potential effects of glycemic status, sex, alcohol consumption, smoking, age, BMI and sequencing run. However, other factors such as diet, lifestyle, medication and technical factors e.g. DNA extraction, were previously shown to affect the oral microbiota^44–46^. Regardless of the adjustment for non-genetic covariates in our analysis, there is a considerable risk that other unassessed factors have confounded the effects of genetics. Additionally, established genetic effects on the oral microbiota in this study may not be direct, but mediated by other mechanisms. To advance towards causal interpretation of our findings, appropriate causal frameworks would have to be developed with carefully chosen adjustment sets for each of the associations.

## Conclusion

In the present study, we uncovered multiple novel variants in the host genome that associate with diversity and abundance of oral bacteria in a Danish prediabetic population with varying risk for T2D. Notably, more of the identified genome variants are also linked to cardiometabolic traits, indicating potentially overlapping genetic factors. Findings of this study and the provided data may facilitate future larger studies and meta-analyses comprising various population profiles to achieve a fuller picture of the host-oral bacterial microbiota interactions.

## Methods

### Study population

The ADDITION-PRO study (2009–2011)^27^ is a risk-stratified -cohort designed to explore T2D and cardiovascular disease risks and their underlying mechanisms among Danish adults. ADDITION-PRO is a subsidiary study of the Danish arm of the ADDITION trial (baseline: 2001–2006) which assessed the effect of intensive multifactorial cardiometabolic treatment on incident cardiovascular events in people with screen-detected diabetes^47^. The general-practice based screening programme used to identify the participants of the trial^48^ also identified a large group of people in various pre-diabetic and high-diabetes risk states; these participants constitute the sampling frame for ADDITION-PRO (2009–2011). The study was approved by the Scientific Ethics Committee in the Central Denmark Region (20000183). All recruited participants gave their written consent for participation in the study. The study was conducted in accordance with the principles of the Declaration of Helsinki.

In total, 786 participants with varied glycemic status were recruited from the Copenhagen study site and provided saliva samples. Participants have undergone an extensive clinical examination including anthropometrics and detailed characterization of glycemic status, as described in detail elsewhere^27^.

### Sample collection

A detailed description of saliva sample collection has been provided in a recent publication^34^. In brief, saliva was collected from 786 participants at Steno Diabetes Center Copenhagen, Copenhagen, Denmark. Participants were asked to refrain from brushing teeth on the day of the health assessment. Saliva production was stimulated with paraffin wax, and collected samples of whole saliva were stored immediately at − 80 °C.

### Saliva sample processing

DNA extraction of saliva samples and 16S rRNA gene amplicon sequencing was done as reported^34^. Microbial DNA was isolated using the NucleoSpinSoil kit (Macherey–Nagel, Germany). Bacterial cells were lysed using SL1 + Enhancer buffer SX. DNA yield and purity were assessed using a Qubit 2.0 flourometer, and a NanoDrop 2000 spectrometer (Thermo Fisher Scientific Inc., MA, USA), respectively. Genomic DNA in each sample was normalized to 30 ng prior to PCR amplification of the 16S rDNA V4 hypervariable region. The PCR products were purified with the AmpureXP kit. The quality and quantification of the final library was determined using the 2100 bioanalyzer instrument (Agilent, DNA 1000 Reagents), and by real-time quantitative PCR (EvaGreenTM).

All samples were consecutively processed at the same location and with the same equipment. Owing to the sample number, sequencing was carried out in two runs (hereafter referred to as R1 and R2) using paired-end 500 cycles sequencing chemistry on an Illumina HiSeq 2500 platform, generating a total of 29,225,397 reads in the first run (R1, n = 666) and 4,193,449 reads in the second run (R2, n = 120).

### 16S rRNA gene amplicon sequence processing

Processing of raw sequencing data was done as reported in a recent publication^34^. In short, raw sequence data were processed using the dada2 algorithm v1.19.1^49^, implemented in metabaRpipe^50^. Following truncation and filtering of raw sequence reads, error rate learning was performed, and amplicon sequence variants (ASVs) were inferred using a pseudo-pooling strategy. Forward and reverse reads were then merged with a minimum overlap of 40 nucleotides. The previous steps were carried out separately for R1 and R2. ASV tables were then merged, and chimeric sequences were identified and removed. A final clustering step was performed to detect potential batch-specific artefacts and no ASVs were clustered confirming the high sensitivity of the approach.

Taxonomical classification of sequences was performed using the dada2 algorithm and the eHOMD database (version V15.22, https://www.homd.org). A phylogenetic tree of ASV sequences was constructed using the phyloseq package v1.22.3^51^.

### Genotyping

Genotyping of 1657 participants from the ADDITION-PRO study^27^ was performed using the Illumina Infinium HumanCoreExome Beadchip (Illumina, San Diego, CA, USA) as described previously^52^. Genotypes were called using the Genotyping module (version 1.9.4) of GenomeStudio software (version 2011.1, Illumina). After filtering out first and second-degree relatives, duplicates, individuals identified as ethnically non-European outliers, individuals with extreme inbreeding coefficients, or mislabelled sex, 1548 individuals passed quality control criteria, 610 of whom had provided saliva samples. Hardy–Weinberg Equilibrium filter with a significance threshold of 0.005 was applied to ensure the accuracy and reliability of the genotyping data. Imputation was done using the Michigan Imputation Server^53^, resulting in a total of 39,117,105 SNPs which were either genotyped or imputed. Genetic principal components (PCs) were computed using plink v1.90^54^.

### Association of oral bacterial features with host genetics

Associations of individual bacterial taxa were tested adapting a previously described workflow^55,56^. In short, univariate bacterial features including ASVs and taxonomic groups from genus to phylum were tested. Taxonomic groups were derived using *tax_glom()* function from the phyloseq R package^51^. Only taxa that were present in more than 100 participants, with median sequence count above 50 were kept for the analysis. To minimize redundancy, taxa that remained after filtering were clustered together based on similarity, evaluated by a Spearmann correlation cut-off of 0.985. This resulted in 43 clusters (Fig. S1). From each cluster, bacterial features from the lowest taxonomic group were selected for univariate tests: two phyla, one class, four order, five families, 12 genera and 19 species (Fig. S2, Table S1).

Statistical association testing between identified bacterial taxa and host genotypes was performed, using R version 4.1.2, following previously described workflow^56^. In short, zero-truncated non-rarefied count abundances were fit in generalized linear models with negative binomial distributions as predictors, with the function *manyglm()* from the Mvabund R package^57^. The following covariates were included to the model: sex, age, BMI, smoking, alcohol, glycemic state, sequencing run, and the first 10 genetic principal components. The logarithm of the total sequence counts of each sample was included to the model as offset. The residual variation from each univariate feature of this model, extracted using the *residuals()* function, was regressed on the host genotype variants (coded into numeric features, 0 = homozygous for reference allele; 1 = heterozygous; 2 = homozygous for alternative allele) in a linear model. Only common SNPs that had a minor allele frequency (MAF) > 5%, and a call rate > 95% were used. *P-*values were obtained from the *summary()* function.

To assess the impact of genetic variants on the overall bacterial community differences (beta diversity), we performed a distance-based F test (DBF-test), as previously described by Rühlemann et al.^28^. Bray–Curtis dissimilarities were computed from normalized microbiome counts (using *rarefy_even_depth()* function, 5000 sequences per sample), using the *distance()* function from the phyloseq R package^51^. Prior to analysis, Bray–Curtis dissimilarity matrix was corrected for covariates sex, age, BMI, smoking, alcohol, glycemic state, sequencing run, and the first 10 genetic principal components. The correction was done by fitting a distance-based redundancy model (*capscale()* function of the vegan R package) and selecting the aforementioned covariates to be partialled out. The residual variation of this model was subsequently used for the DBF-test. Only SNPs that had a MAF > 5%, imputation QC > 0.6 and call rate > 95% were used.

*P*-values that passed the genome-wide significance threshold of *P* ≤ 5 × 10^−8^ are reported as statistically significant. *P*-values meeting the study-wide significance threshold of *P* ≤ 1.19 × 10^−9^, derived by dividing the standard genome-wide significance level of *P* = 5 × 10^−8^ by the number of GWAS tests performed in this study (n = 44), reported as study-wide significant. For each GWAS, we grouped genetic variants that reached significance in 1 Mb genomic regions, assigned the strongest genetic association as lead variant, and named the locus based on the nearest gene.

The local linkage disequilibrium (LD) between the SNPs in the significantly associated loci was estimated in plink v1.9^54^ and visualized in R using a publicly available script (https://github.com/Geeketics/LocusZooms).

Manhattan- and QQ-plots were generated and variants were annotated using the topR package^58^.

Summary statistics of suggestive and significant findings from univariate tests (Table S2), and full summary statistics for each of the performed test are available in the supplementary material and GWAS catalog.

### Associations between identified SNPs and common oral and metabolic traits

Variants, identified by GWAS as being significantly associated to univariate and multivariate bacterial features, were queried in publicly available European ancestry GWAS datasets of blood pressure, cardiometabolic disease, glycemic traits, oral health traits and traditional lipids^59–64^(Table S3). The phenotype sets were selected based on the findings from previously published observational associations between oral microbiota composition and lifestyle traits in the same cohort^34^. Z scores (beta/standard error) were extracted from all summary statistics and aligned to the effect allele. The *P*-value was considered suggestive at 0.05 and significant at 0.05 after FDR correction^65^.

### Ethical approval

The ADDITION-PRO study was approved by the Scientifics Ethics Committee in the Central Denmark Region (20000183). All studies were conducted in accordance with the principles of the Declaration of Helsinki.

### Supplementary Information

Supplementary Figures.

Supplementary Table S1.

Supplementary Table S2.

Supplementary Table S3.

Supplementary Table S4.

## Data Availability

16S rRNA sequencing data for the full Addition-PRO study (n = 745) has been deposited in the European Nucleotide Archive (ENA) at EMBL-EBI under accession number PRJEB57196 (https://www.ebi.ac.uk/ena/browser/view/PRJEB57196). GWAS summary statistics (N = 44) have been uploaded to EMBL-EBI GWAS catalog^66^ under study accession numbers GCST90429799-GCST90429842. The raw data are not publicly available due to privacy or ethical restrictions.
