# Supplementary material for: Genome-wide association study identifies host genetic variants influencing oral microbiota diversity and metabolic health
Source: Sci Rep. 2024 Jun 26;14:14738. doi: 10.1038/s41598-024-65538-8 (PMC11208528; doi:10.1038/s41598-024-65538-8)
Supplement: Supplementary file 1 — Supplementary Figures. [file 41598_2024_65538_MOESM1_ESM.pdf]

GENOME-WIDE ASSOCIATION STUDY IDENTIFIES HOST GENOME VARIANTS AFFECTING THE COMPOSITION OF THE SALIVARY MICROBIOTA

Evelina Stankevic[1], Timo Kern[1], Dmitrii Borisevich[1], Casper Sahl Poulsen[1], Anne Lundager Madsen[1], Tue Haldor Hansen[1,2], Anna Jonsson[1], Mikkel Schubert[1], Nikoline Nygaard[7], Trine Nielsen[1,2,3], Daniel Belstrøm[7], Tarunveer S Ahluwalia[5,6], Daniel R. Witte[4,8], Niels Grarup[1], Manimozhiyan Arumugam[1], Oluf Pedersen[1,9], *Torben Hansen[1]

1 Novo Nordisk Foundation Center for Basic Metabolic Research, Faculty of Health and Medical Sciences, University of Copenhagen, Copenhagen, Denmark

2 Medical Department, Zealand University Hospital. Koege, Denmark

3 Department of Clinical Medicine, University of Copenhagen, Denmark

4 Department of Public Health, Aarhus University, Aarhus, Denmark

5 Steno Diabetes Center Copenhagen, Herlev, Denmark

6 The Bioinformatics Center, Department of Biology, University of Copenhagen, Copenhagen, Denmark

7 Department of Odontology, Section for Clinical Oral Microbiology, University of Copenhagen, Copenhagen, Denmark

8 Steno Diabetes Center Aarhus, Aarhus, Denmark

9 Center for Clinical Metabolic Research, Herlev-Gentofte University Hospital, Copenhagen, Denmark

***Corresponding author:** Torben Hansen, Torben Hansen, The Novo Nordisk Foundation Center for Basic Metabolic Research, Faculty of Health and Medical Sciences, University

of Copenhagen, Blegdamsvej 3B, DK-2200 Copenhagen N, Denmark. Email:

torben.hansen@sund.ku.dk

Competing interests:

The authors declare no competing financial interests.

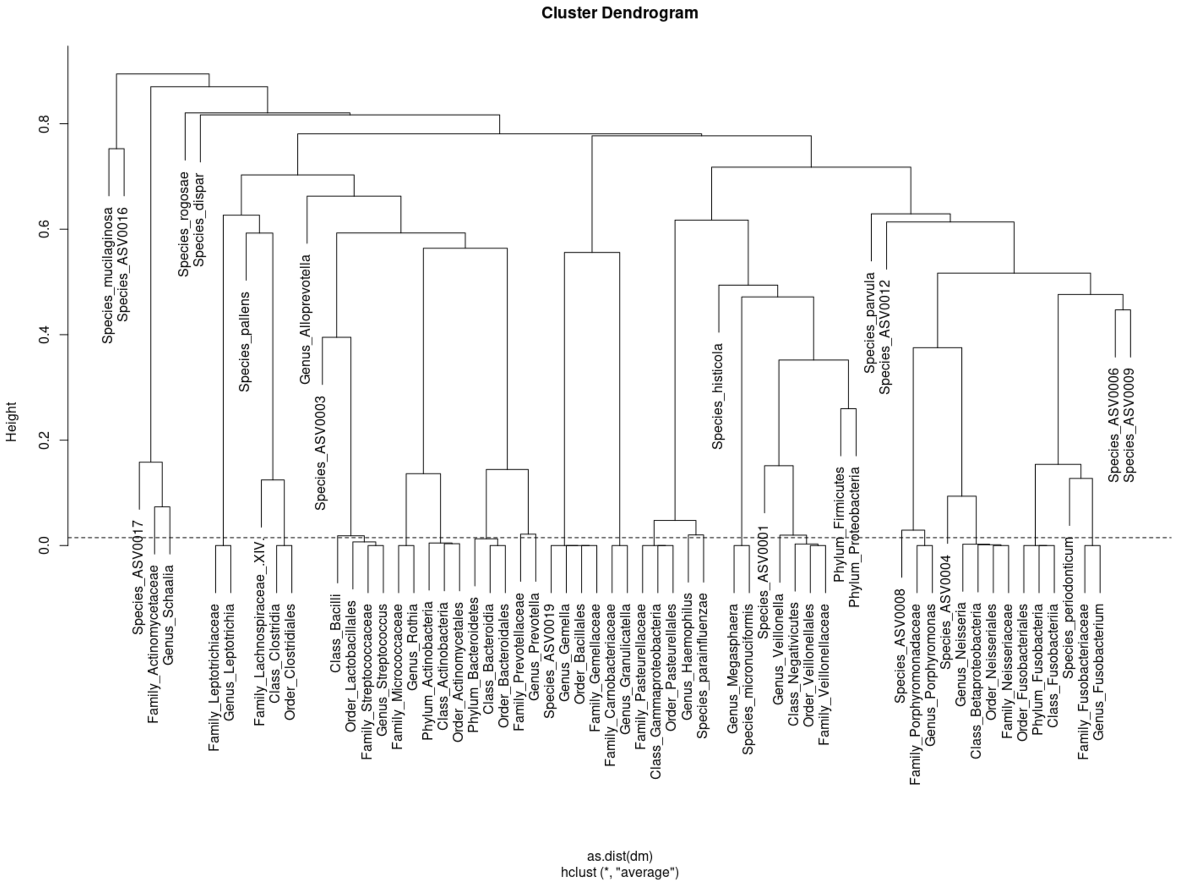

Figure S1. Dendrogram of univariate bacterial features, clustered by similarity. Univariate bacterial features were clustered together based on 0.985 Spearman correlation cut off value. Dashed line represents the similarity cutoff at 0.985. This effort resulted in 43 distinct clusters from 110 univariate bacterial features.

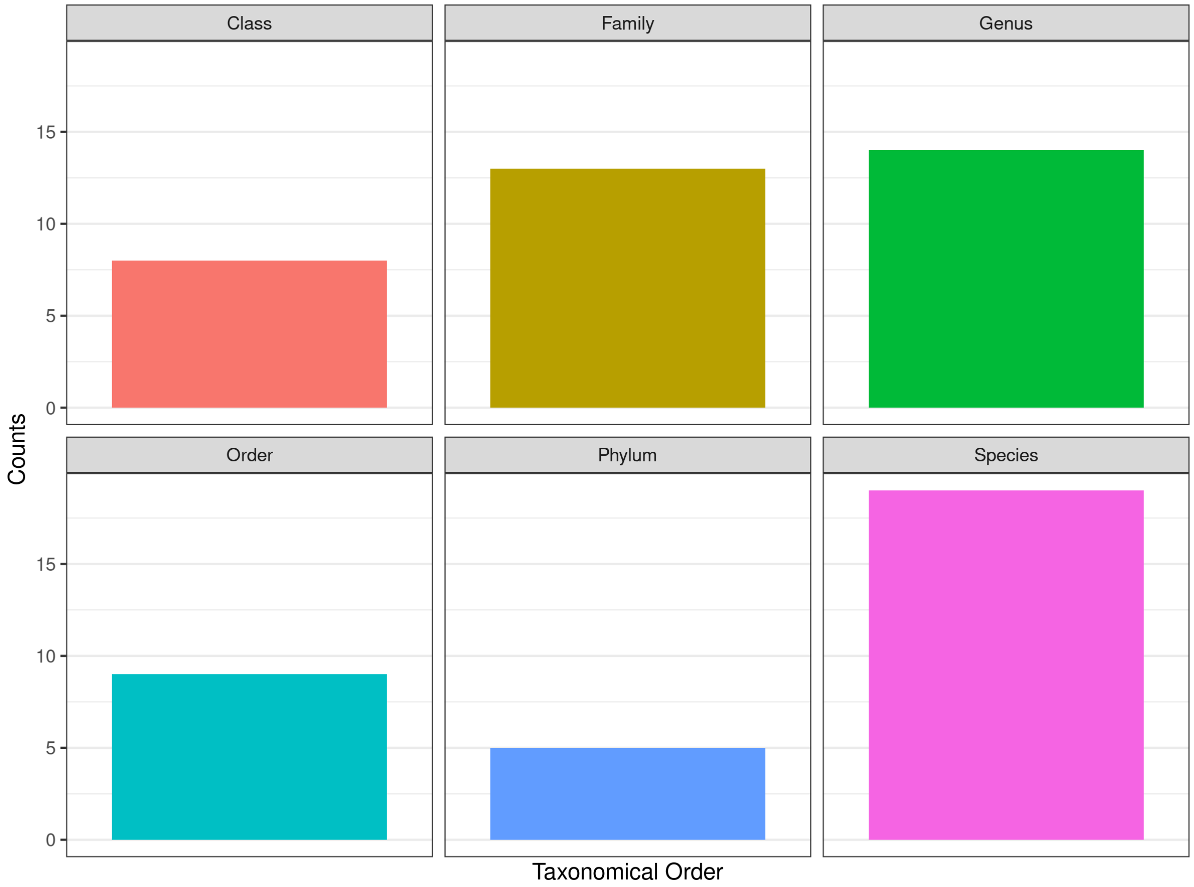

Figure S2. Count of tested univariate bacterial features, stratified by bacterial genera. The box plot visualises the number of univariate bacterial features tested per each of bacterial genera.

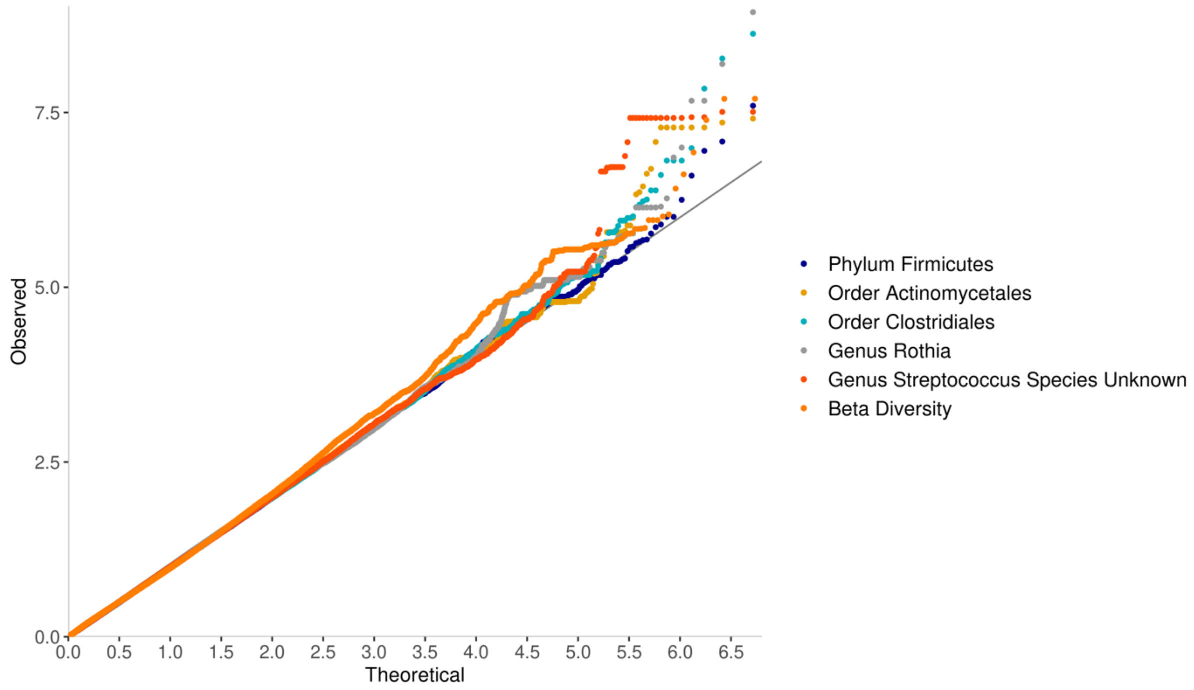

Figure S3. QQ plot representing results from GWAs on oral microbial features.
The color-coding represents 6/44 bacterial features that had variants reaching genome-wide significance level ($P < 5 \times 10^{-8}$).
